# White matter changes in patients with mild traumatic brain injury: MRI perspective

**DOI:** 10.2217/cnc-2016-0028

**Published:** 2017-03-22

**Authors:** Ponnada A Narayana

**Affiliations:** 1 Department of Diagnostic & Interventional Imaging, McGovern Medical School, University of Texas Health Science Center at Houston, Houston, TX 77030, USA

**Keywords:** MRI, MRI–pathology correlation, MRS, mTBI, WM injury

## Abstract

This review focuses on white matter (WM) changes in mild traumatic brain injury (mTBI) as assessed by multimodal MRI. All the peer reviewed publications on WM changes in mTBI from January 2011 through September 2016 are included in this review. This review is organized as follows: introduction to mTBI, the basics of multimodal MRI techniques that are potentially useful for probing the WM integrity, summary and critical evaluation of the published literature on the application of multimodal MRI techniques to assess the changes of WM in mTBI, and correlation of MRI measures with behavioral deficits. The MRI–pathology correlation studies based on preclinical models of mTBI are also reviewed. Finally, the author's perspective of future research directions is described.

About 2% of the US population is affected by traumatic brain injury (TBI) which results in an annual healthcare cost of $76.5 billion [1]. Based on clinical symptoms, TBI can be categorized as either mild or moderate or severe [2]. Focal TBI generally results in contusion or laceration to cortical and subcortical structures and intracranial bleeding and results in severe TBI. In contrast, diffuse injury is the result of stretching and tearing of axons due to linear and angular acceleration/deceleration. Linear acceleration causes less severe axonal shearing than angular acceleration/deceleration [3]. Traumatic axonal injury (TAI) is thought to be a major pathologic event in TBI and may be responsible for much of the observed cognitive deficits [4–12]. Diffuse axonal injury (DAI) may be considered as a subset of TAI. Diffuse axonal injury has some histopathological characteristics that occur immediately and within hours and days of injury. Axons in white matter (WM) are vulnerable to diffuse injury because of their small diameter (∼3 μ) and long projections. DAI could result in mechanical breaking of the axonal cytoskeleton that could affect axonal transportation which in turn affects communication between different brain structures. WM pathology, and especially that detected with neuroimaging, is also influenced by other factors such as blood flow/edema, metabolic aberrations, excitotoxicity and secondary degeneration, Wallerian degeneration, and other factors, which occur under the umbrella of TAI and coalesce over time, not just what occurs within the acute time frame. Depending on the severity of DAI, TBI victims could experience cognitive and behavioral deficits. Advances in biomechanical modeling identified WM pathways that are most vulnerable to stretch and shear injury. These pathways include corona radiata (CR), internal capsule (IC), cerebral peduncle (CP) and corpus callosum (CC) [13–15].

About 80% of TBI incidents in military and civilian populations are classified as mild (mTBI) [16], which is considered to be a major public health problem [17–19]. mTBI could result from any number of mechanisms that include blunt trauma resulting from falls and vehicular accidents, collisions such as those experienced in sports related activities, and blast, experienced mainly by army personnel. The diagnostic criteria for mTBI typically include loss of consciousness (LOC) for less than 30 min, amnesia for a period of less than 24 h, and a score of 13–15 on the Glasgow Coma Scale [20]. Majority of the post-mTBI symptoms are resolved spontaneously within days to a few weeks. In about 30% of mTBI patients postconcussion symptoms (PCS) persist over a period of several months or longer [21]. TRACK-TBI study indicates that about 22.4% of the mTBI subjects are not completely functional at 1 year post injury [22]. Notwithstanding the word ‘mild’, mTBI patients experience somatic, affective, and cognitive symptoms, collectively referred to as PCS or postconcussional disorder that compromises the quality of life. Understanding the reasons for the prolonged PCS in these patients has important implications in the management of mTBI.

Concussion has recently attracted considerable attention because of the injuries suffered by high school, college and professional players [23,24], and military personnel [25]. Concussion is generally considered to be the mildest form of mTBI. Concussion and mTBI have similar and overlapping symptoms [26]. As pointed out elsewhere ‘all concussions are mild TBIs, though all mTBIs are not concussions’ [27]. Nevertheless, these two terms are used interchangeably in the published literature. In this review, consistent with most published literature, the terms concussion and mTBI are used interchangeably. In this review, whenever possible, we tried to follow the recommended definition of the postinjury phase: acute <1 day; subacute: 1 day–1 week; postacute 1 week–6 months; chronic: >6 months [28].

The commonly used techniques for evaluating WM injury in mTBI include immunohistochemistry, electron microscopy and neuroimaging. The first two techniques require tissue samples and are not practical for routine use in clinical studies. The commonly used noninvasive neuroimaging techniques for evaluating injury in mTBI include computerized tomography (CT) and MRI. Among these two, MRI is most commonly used for evaluating mTBI because of its superior soft tissue contrast and its multimodal nature that can provide anatomical, structural, functional, physiological and metabolic information [29–32]. MRI also has the ability to detect subtle pathology in mTBI even when the clinical symptoms appear to return to normal values [30,33–36]. Conventional MRI based on T1-, T2-weighted and FLAIR images is of limited use in detecting WM injury in mTBI [29,37,38]. As discussed below, published literature also indicates the potential of more recent MRI-based techniques that include, diffusion tensor imaging (DTI), magnetization transfer ratio (MTR), susceptibility weighted imaging (SWI), myelin water imaging, ultrashort echo time, and proton magnetic resonance spectroscopy (MRS) in detecting WM injury in mTBI. Among these, DTI has shown considerable promise in identifying underlying neuropathology in mTBI [29]. This review does not cover functional MRI (fMRI) [39] or positron emission tomography (PET) [40] since the focus of these modalities is mainly on gray matter.

The role of advanced neuroimaging techniques in TBI were recently reviewed [41]. The TRACK-TBI study also demonstrated the superiority of advanced MRI in predicting outcome in mTBI at 3 and 6 months postinjury time [42]. The published literature on WM injury in human brain using different MRI-based techniques is summarized in Table 1. Since prior reviews summarized published literature till 2011, we included only publications listed in Pubmed from January 2011 through September 2016. Inclusion of publications in 2011 provides an overlap with the previous reviews. We also summarize in Table 2 the review articles on mTBI published from January 2011 to September 2016 with a strong neuroimaging component. The basics of these techniques are briefly reviewed, followed by their application to WM injury in mTBI.

**Table 1.** **Publications on white matter changes in human mild traumatic brain injury since 2011.**

**Number of subjects**	**Age (years)/(F/M)**	**Postinjury time**	**Field strength (T)**	**Technique/analysis method**	**Affected/measured WM regions**	**Major conclusions**	**Ref.**
m: 23 (11 [GO]/12 [PO]) C: 23	C:30 ± 8.4 (11/12); GO: 27.8 ± 8.5 (3/8) PO: 31.3 ± 8.4 (5/7)	Between 7 and 28 days and between 3 and 4 months	1.5	DTI/TBSS	Increased MD in CC, the right ATR and the SLF, the ILF and the FOF bilaterally	DTI at the subacute stage may be a predictive marker of poor outcome	[43]

m: 60 (with PCD: 21; without PCD: 39) C(OI): 32	Between 19 and 55 m: (17/43) OI: (9/25)	6–8 weeks	3	DTI/ROI	MD of SCC	Does not support an association between WM integrity in the CC and self-reported PCS 6–8 weeks post mTBI	[44]

m: 25 (veterans exposed to BI) C: 33	BE: 24–55 No BE: 22–52 (4% females)	2–5 years	3	DTI/Voxel-wise	Forceps major and minor, bilateral anterior thalamic radiations, right corticospinal tract, bilateral IFOF, bilateral ILF and left SLF	Blast mTBI disrupts integrity of number of white matter tracts; these disruptions are diluted by averaging across large number of voxels within an ROI; the neurological effects of blast mTBI are diffuse, widespread and spatially variable	[45]

m: 34 C: 30	m: 29.9 ± 6.4 (M); (19/15) 38.9 ± 13.2 (F); C: 36.6 ± 11.9 (M); 38.1 ± 10.3 (F) (14/16)	<2 weeks, 3 and 6 months	3	DTI/enhanced Z-score micro-structural assessment for pathology (EZ-MAP; voxel-wise analysis)	High FA most frequently detected in the deep and subcortical white matter of the frontal, parietal and temporal lobes, and in the anterior portions of the CC Low FA values were observed in CR (anterior and superior), SCC, precentral white matter, IC and deep and subcortical WM	Unique spatial patterns of WM abnormalities in each patient Implications of high FA remain unclear, but may be evidence for a compensatory mechanism or plasticity in response to injury, rather than a direct manifestation of brain injury	[7]

m: 8 C: 0	Between the ages of 18 and 40 (3/5)	<48 h	3	DTI/ROI based on tractography	Cingulum	Memory performance appeared to mirror changes in FA in certain cases, supporting a pathophysiological basis to memory impairment following mTBI	[46]

m: 5; C: 50	m: (37.1 ± 10.2) (33/28) C: (35.8 ± 13.4) (20/30)	At least 6 months	1.5	DTI/TBSS	Right SLF, left superior frontal gyrus, right insula and left fornix	In the chronic stage certain regions have abnormally reduced WM integrity; these brain regions perhaps are related to chronic persistent cognitive impairments	[47]

m: 14 (4 with MDP and 10 without MDP) C: 0	MDP: 37 ± 9 (0/4) W/O D: 35.7 ± 12 (4/6)	MRI within 1 month	3		WM abnormalities in the frontotemporal regions in the depressed group	Compared with the nondepressed group, those who developed depression had white matter abnormalities	[48]

m: 46 (22 with LOC; 24 with AOC) C: 0	LOC: 29.1 ± 6.07 AOC: 26.5 ± 4.92 (0/46)	Not indicated	3	DTI HARDI acquisition/TBSS analysis	FA is significantly lower in LOC compared with AOC in bilateral brainstem, inferior longitudinal fasciculus, inferior fronto-occipital fasciculus, body of the CC, cingulum, SLF and anterior thalamic radiations	Evidence of microstructural alterations in individuals with history of LOC, and may suggest brain basis for psychiatric symptoms and mental illness following mTBI	[49]

m: 14 C: 14	m: 34.9 ± 18.4 (5/9) C: 35.8 ± 18.5 (5/9)	2 days (12–72 h) and 1 month (28–43 days)	3	DTI/TBSS	Anterior CC, right CR and IC	TBSS showed fractional anisotropy (FA) to be significantly lower, and mean diffusivity (MD) to be higher in the mTBI group in several white matter tracts (FA = 40,737; MD = 39,078 voxels) compared with controls at 72 h after injury and still 1 month later for FA. Results underscore the importance of strictly defined image acquisition time points when performing MRI studies on patients with mTBI	[50]

m: 26; C: 13	m: 30 ± 11(5/25) C: Not indicated	3–55 days	3	MRS/Global	PCS-positive patients (n = 15) had lower WM NAA than the controls (n = 12)	Global WM NAA, showed sensitivity to the TBI sequelae associated with common PCS	[9]

m:20 (at base line) and 10 at follow-up; C: 16	m: 34.8 ± 10.7 (4/16 at baseline 2/8 at follow-up) C: 35.1 ± 11.9 (3/13)	Within 1 month and >9 months	3	DTI and DKI/ROI	IC, EC, CC, cingulum, CSV at baseline. CC, cingulum, optic radiations and CSV at follow-up	DTI and DKI, I might be useful for investigating dynamic changes in WM and cognitive impairment during a short follow-up period	[51]

m: 11 with PPCS; C: 11	m: 32.1 ± 8.5 (2/9) C: 32.1 ± 8.5 years (1/10)	62.08 ± 46.3 months	3	DTI/ROI	CC	Individualized analysis shows promise for enhancing clinical care of PPCS patients as it could play a role in the diagnosis of brain injury not revealed using conventional imaging	[52]

m: 11 C: 11	m: 15.09 ± 1.14 (6/5) C: 15.82 ± 1.78 (6/5)	1–6 days	3	DTI/ROIs on fiber tracts	Fornix	Relation between lower performance on cognitive tasks and higher FA in fornix	[53]

m: 34 C: 42	m: 34.9 (not indicated) C: 38.3 (20/22)	<2 weeks	3	DTI/Voxel wise analysis (FMRIB diffusion toolbox)	Multiple WM regions, depending on individual patient	EZ-Map was used to provide robust approach for detecting abnormal FA in individual patients	[54]

m: 34 (with blast) C: 18 (without blast)	Blast: 31.6 ± 9.2 (0/34) nonblast: 32.8 ± 7.3 (1/17)	Not indicated	3	DTI/Voxel-wise analysis MPF based on MTR	Reduced FA in right genu of the CC in blast vs nonblast veterans; lower MPF in right genu of the CC, capsule-anterior limb); the interlobar right SLF; frontal and parietal subgyral WM (i.e., right precentral, superior white matter hyperintense lesions on FLAIR sequence were observed in mTBI [32]. In veterans with mTBI, it was shown that these lesions have an effect on verbal memory, independent of the presence of PTSD and middle frontal gyri, medial parietal gyrus/precuneus and left superior parietal lobule); frontal GM/WM border regions (i.e., right superior and middle frontal and left inferior frontal gyri	Veterans with one or more blast-related mTBIs exhibit abnormalities of brain WM structural integrity and macromolecular organization that are not related to comorbid PTSD	[55]

m: 30 (decreased EF: 13; without decreased EF:17) C: 15	m: 30.7 ± 9.3 (87% M); C; 32.9 ± 8.2 (73% M)	Not indicated	3	DTI/ROI based on TBSS	Participants with EF decrements demonstrated significantly decreased FA in prefrontal white matter, CC and cingulum bundle structures compared with mTBI participants without EF decrements	LOC may be a risk factor for reduced EF as well as associated changes to ventral prefrontal white matter	[56]

m: 21 C: 22	m: 33.2 ± 12.4 (2/19) C: 31.5 ± 11.7 (2/20)	Within 3 days	1.5	MRS/ROI	Significant decrease in NAA was found in both frontal lobes and in NAA/Cre ratio in the right frontal lobe; lower NAA was found in upper brainstem in the subgroup of patients with post-traumatic unconsciousness; regions contain both GM and WM	Correlation between metabolite changes and cognitive decline and presence or absence of loss of consciousness in acute phase	[57]

m: 103 (ES: 43, LS: 33, Ch: 27); C: 21	m(ES): 40.63 ± 17.31(10/43); m(LS): 37.64 ± 16.60 (10/43) Ch: 40.11 ± 17.33 (9/27) C: 39.76 ± 18.04 (8/21)	ES: 5.44 ± 3.15 days LS: 37.00 ± 12.26 days Ch: 195.30 ± 19.60	3	MRS/3D PRESS	Reduced Cho/Cr in Subacute phase in CSV; positive association of Cr (in ES) in the centrum semiovale with chronic automated neuropsychological assessment metrics	Metabolic measurements in centrum semiovale can potentially serve as diagnostic and prognostic markers in mTBI	[58]

m: 76 (44 without intracranial lesions; 32 with intracranial lesions: 32) C: 50	Without lesions: 31.2 ± 9.5 (17/27); with lesions: 33.9 ± 12.0 (9/23) C: 28.7 ± 9.2	11.2 ± 3.3 days	3	DTI/Voxel-wise and ROI	Lower FA in GCC, UF and anterior CR bilaterally as well as right IC and EC in patients with intracranial lesions compared with controls; no difference in the DTI parameters between subjects with an without intracranial lesions	For subset of patients lacking neuropsychiatric and substance abuse history, MRI surpassed all other predictors for both 3- and 6-month outcome	[42]

m: 21 (with concussion) C: 16	Concussed: 20.19 ± 1.03 (0/21) C: 19.9 ± 1.67 (0/16)	2 days, 2 weeks and 2 months	3	DTI/TBSS	Regions implicated are all in right hemisphere: posterior limb of the IC, retrolenticular part of IC, sagittal stratum (ILF, IFOF) and ATR	Support the hypothesis of increased RD and reduced FA within 72-h post injury, followed by recovery that extended beyond 2 weeks; RD appears to be sensitive measure of concussive injury	[59]

m: 69; C: 21	m: M: 18; range of 10–38; F: 16.7 with range of 12–25 (22/47); C: M: 17 with range of 6–42; F: 17 with range of 7–44 (11/10)	Not indicated	1.5	DTI/TBSS	Lower FA value in in males compared with females	Relative sparing of the UF is seen in females compared with male patients, with sex and FA in UF as stronger predictors of TSR than initial symptom severity	[60]

m: 62 C: 59	m: 30.4 ± 8.8 (17/45) OI: 29.2 ± 9 (14/45)	27.1 ± 13.7 h and 97.9 ± 17.57 days	3	DTI/TBSS MTR MRS based on 2D phase encoding	At baseline, MD was significantly higher in mTBI cohort relative to comparison group in several WM regions that included IC, superior CR, anterior CR, posterior CR, IFOF, ILF, forceps major and forceps minor of CC, SLF and CST in the right hemisphere MTR, MRS and volumetry did not show significant differences between groups and at different time points in the same group	Number of WM tracts are affected in mTBI in acute phase of injury and these changes disappear by 90 days none of the MRI modalities used in this study, with the exception of DTI, is sensitive in detecting changes in the acute phase of mTBI	[30]

m: 36 OI: 37	m: 29.0 ± 8.4 (13/23) OI: 29.4 ± 9.2 (10/27)	∼24 h and ∼90 days	3	DTI/ROI based on atlas Volumetry	Elevated MD in CR	Potential utility of DTI to capture transient edema in CR	[61]

m: 45 (38: neuropsychiatric symptoms; 32: irritability; 32: depression; 18: anxiety. Of these, 13 patients had only irritability, one had only depression, 14 had comorbid irritability and depression, seven had comorbid anxiety and depression, and one had comorbid irritability and anxiety. Ten patients fulfilled the criteria for all three conditions) C: 29	m: range 11–47 (11/27) C: 10–28 (8/21)	Median 20 days (range 0–506 days)	1.5	DTI/ROI based on TBSS	Compared with controls, mTBI patients with depression had decreased FA in the superior longitudinal fasciculus, WM around the nucleus accumbens and anterior limb of IC	Detection of the central white matter injuries that underlie depression and anxiety, but not irritability, indicates that not all neuropsychiatric symptoms after mTBI are result of discrete white matter injuries	[62]

m: 59 (31: with LOC: 28: without LOC) C: 55 (blast exposed without mTBI)	M (without LOC: 29.6 ± 7.7) (1/30); with LOC: 27.9 ± 4.2 (0/28); C: 30.5 ± 6.7	m: (without LOC): 12.6 ± 12.2 months m (with LOC): 7.2 ± 8.6 months C: 9.4 ± 11.7 months	3	DTI/appears like voxel-wise analysis	Reduced FA in left retrolenticular part of the internal capsule	These results support postmortem reports of diffuse axonal injury following mTBI and suggest that injuries with LOC involvement may be particularly detrimental to white matter integrity These results also suggest That LOC-associated WM abnormalities influence neurocognitive function	[63]

m: 38 C: 20	m: 33.37 ± 6.44 (5/33) C: 29.35 ± 5.49 (10/10)	m: 90.92 ± 48.23 days	3	DTI/tractography	No significant differences in FA between the C and m groups in brainstem white matter tracts (i.e., medial lemniscus-central tegmentum); CST, and pontine tegmentum	Collectively, these data point to important neurobiological substrates of the chronic and complex constellation of symptoms following mTBI in veterans	[64]

m: 23 C: 20 (OI)	m: 13.2 ± 1.8 (2/21) C: 12.7 ± 1.5 (5/15)	m: 45.0 ± 17.6 h OI: 48.2 ± 21.1 h	3	Voxel-wise analysis	m group had significantly higher FA and AD in middle temporal gyrus WM, superior temporal gyrus WM, anterior CR and SLF; m group had also significantly lower MD and/or RD in a few WM regions including the middle frontal gyrus WM and anterior CR	Alterations of diffusivity in spatially heterogeneous WM regions shortly after mTBI in youth may reflect restrictive water diffusion in WM early post injury	[65]

m:40 C: 50	m: 38.03 ± 13.69 (16/24) C: 29.88 ± 10.75 (12/38)	74.43 ± 103.37 h	3	DTI/tractography	Major WM tracts include CC, and SLF and ILF	Increased interactions among action–emotion and action–cognition as well as within perception networks; suggests that mTBI may result in changes of structural and functional connectivity on a connectome scale at the acute stage	[66]

m: 11 (sustained concussion during the study) C: 24	21.2 ± 3.1 (20/25)	72 h, 2 weeks, 2 months	3	Myelin water imaging	Reduction in myelin water fraction at 2 weeks post injury relative to preseason scans in the SCC, right posterior thalamic radiation, left superior CR, left SLF and left posterior limb of IC Myelin water fraction recovered to preseason values by 2 months post injury	Indicate transient myelin disruption following a single mTBI, with subsequent remyelination of affected neurons; myelin disruption was not apparent in the athletes who did not experience a concussion, despite exposure to repetitive subconcussive trauma over a season of collegiate hockey; findings may help explain many of the metabolic and neurological deficits observed clinically following mTBI; myelin disruption was not apparent in the athletes who did not experience a concussion, despite exposure to repetitive subconcussive trauma over a season of collegiate hockey	[67]

m: 74 (57 with PTM and 17 without PTM) C: 42 (22 healthy controls and 20 migraine controls)	m: 18 with a range of 10–47(23/51) C: Healthy controls 18.8 with a range of 6–44 (12/10) Migraine controls 21.7 with a range of 16–43 (10/10)	20 days (range: 0–506 days)	1.5	DTI/Whole brain FA histogram and Shanon Entropy	–	SE more accurately reveals mTBI than mean FA, more accurately reveals those patients with mTBI who develop PTM, and inversely correlates with time to recovery	[68]

m: 79 OI: 64	m: 29.6 ± 8.8 (24/55) OI: 28.8 ± 8.5 (17/47)	25.9 ± 12.3 h 94.4 ± 8.7	3	Tractography	FA and MD in left and right UF, left and right IFOF and G CC	LOC was significantly related to MD in UF and IFOF and to FA in left UF and right UF Between-group differences in MD were significant for left UF, left and right IFOF and genu of CC on initial DTI, but not at 3 months post injury; these differences were specific to mTBI subgroup with LOC; early DTI may provide a biomarker for mTBI with LOC, even in patients whose consciousness recovers by arrival in the emergency department; MD better differentiates mTBI from OI than FA on early DTI, but this is specific to mTBI with LOC	[69]

m: 56 (43: passed PVT; 13: failed PVT) C: 23	m:PVT passed: 32.9 ± 8.2 (7/16); PVT failed 31.5 ± 8.5 (1/12) C: 32.9 ± 7.9 (7/16)	m PVT passed: 64.4 ± 43.8 mos; PVT failed: 34.0 ± 20.3 months	3	DTI/tractography	Anterior IC, cingulum and CC	WM abnormalities are evident in those who failed PVTs; poor PVT performance does not negate the possibility of underlying WM abnormalities in mTBI	[70]

m: 3 C: 18 (three groups of 6, one for each m patient)	m: 44.0 ± 9.1 (1/2) C: 54.2 ± 3.9/34.7/43.0 ± 2.9 ± 4.8/(6/12)	2 and 3 months	1.5	DTI/tractography	Inferior cerebellar peduncle	Fiber number of the ICPs decreased by more than 2 SD compared with those of subjects in the control group; evaluation of ICP tractography would be useful in patients with a balance problem	[71]

m: 102 C: 30	m: 47 ± 20 (32/70) C: 50 ± 20; 38.1 ± 10.3 (F) (16/14)	21 ± 15 days	3	DTI/TBSS, single fiber skeleton and whole brain	Decreased FA, increased MD, RD and AD, globally	mTBI is associated with changes in WM; correlation between DTI changes outcome	[72]

M: 25 (sports related concussion) C: 15	m: 21.2 ± 3.1 (0/25) C: 22.9 ± 2.3 (9/6)	72 h, 2 weeks, 2 months	3	FLAR, SWI, volumetric	Small reduction in brain volume at 2 weeks and 2 months; no WMH or microbleeds	–	[73]

AD: Axial diffusivity; AOC: Alteration of consciousness; ATR: Anterior thalamic radiation; BE: Blast exposed; BI: Blast injury; C: Controls; CC: Corpus callosum; Ch: Chronic; Cho: Choline; Cr: Creatine; CR: Corona radiata; CST: Corticospinal tract; CSV: Centrum semiovale; DTI: Diffusion tensor imaging; DKI: Diffusion kurtosis imaging; EC: External capsule; EF: Executive function; ES: Early subacute; F: Female: FLAIR: Fluid attenuation by inversion recovery; FOF: Fronto-occipital fasciculus; GCC: Genu of the corpus callosum; GO: Good outcome; HARDI: High angular resolution diffusion imaging; IC: Internal capsule; ICP: Inferior cerebellar peduncle; IFOF: Inferior frontal occipital fasciculus; ILF: Inferior longitudinal fasciculus; LOC: Loss of consciousness; LS: Late subacute; M: Male; m: mTBI; MD: Mean diffusivity; MDP: Major depression; MPF: Macromolecular proton fraction; MRS: Magnetic resonance spectroscopy; MTR: Magnetization transfer ratio; OI: Orthopedic injured; PCD: Postconcussion disorder; PCS: Postconcussion syndrome; PO: Poor outcome; PPCS: Persistent postconcussive symptom; PRESS: Point-resolved spectroscopy; PTM: post traumatic migraine; PTSD: Post-traumatic stress disorder; PVT: Performance validity test; RD: Radial diffusivity; ROI: Region-of-interest; SCC: Splenium of corpus callosum; SE: Shannon entropy; SLF: Superior longitudinal fasciculus; SWI: Susceptibility weighted imaging; TBSS: Tract-based spatial statistics; TSR: Time to symptom resolution; UF: Uncinate fasciculus; WM: White matter; WMH: WM hyperintense lesion.

**Table 2.** **Reviews and meta-analyses of neuroimaging in mild traumatic brain injury.**

**Comments**	**Ref.**
Reviews the role of neuroimaging in sports-related concussion	[74]

Reviews the implications of DTI findings in mTBI	[75]

Focuses on DTI of frontal lobe	[76]

Focuses on the pathophysiologic role of stretch injury and relation to neuroimaging findings	[8]

Reviews the incidents of mTBI and objective radiological measures	[29]

Meta-analysis of voxel-based DTI	[77]

Reviews DTI application to sports-related concussion	[78]

Reviews DTI application to mTBI	[37]

Summarizes advanced neuroimaging findings in neuropsychological outcome in mTBI	[79]

Reviews neuroimaging studies to highlight the spectrum of acute to chronic time scales and provides meta-analyses to examine the role of MRI for studying both structure and function in mTBI	[80]

Reviews the role of MRS in sports-related concussion	[81]

Summarizes evidence of neurodegeneration following TBI	[82]

Reviews white matter changes in sports related concussion	[83]

Reviews the application of advanced MRI in pediatric TBI	[84]

Reviews the application of high-resolution tractography to investigate white matter injury	[85]

Reviews the role of MRI in TBI	[86]

Reviews the role of, DTI, SWI, fMRI and MRS in mTBI	[31]

Reviews the results of advanced MRI in blast injury	[87]

Reviews the correlation between neuropathology and cognitive and behavioral deficits	[88]

Summarizes the long-term consequences of TBI	[89]

Reviews the application of MRI to mTBI	[38]

Summarizes the results of advanced MRI in mTBI	[90]

Reviews the role for neuroimaging in concussion	[26]

Meta-analysis of voxel-based (TBSS) in mTBI	[91]

Reviews the pitfalls of DTI in mTBI	[92]

Reviews the role of neuroimaging in youth sports	[93]

Provides a summary of clues to white matter injury repair	[94]

Reviews the MRI and CT findings in mTBI, including sports-related concussion	[32]

Summarizes DTI–PCS correlation	[95]

Summarizes the application of multimodal neuroimaging in mTBI	[96]

DTI: Diffusion tensor imaging; fMRI: Functional MRI; mTBI: Mild traumatic brain injury; MRS: Magnetic resonance spectroscopy; PCS: Postconcussion syndrome; SWI: Susceptibility weighted imaging; TBSS: Tract-based spatial statistics.

The pathophysiology of mTBI at the cellular level can be found in a number of excellent reviews [3,97–99] and will not be included here. Since the main focus of this review is on WM, the effect of mTBI on cortical and subcortical structures is not included in this review. Also the effect of repetitive concussions will not be explicitly discussed. Consistent with this journal's recommendation, only references to the works published in the last 5 years or so are included. Thus many important original publications that appeared earlier than 5 years are not referenced. There are a number of excellent reviews that covered earlier studies and references to these review articles are provided at the appropriate place. All relevant publications between January 2011 and September 2016 were identified by searching PubMed using various queries that are combinations of the key words, ‘mild traumatic brain injury’, ‘traumatic brain injury’, ‘concussion’, ‘sports concussion’, ‘MRI’, ‘MRS’, ‘DTI’, ‘white matter hyperintensities and pathology’. Both human and preclinical studies were included in this search.

## MRI techniques relevant to investigate WM injury in mTBI

A number of MRI-based modalities have been used to probe the WM injury in mTBI and to determine possible association between the MRI measures and cognitive, behavioral, and neuropsychiatric disorders in mTBI [38,62,95,100]. Such an association could be helpful in the management of mTBI victims.

### Diffusion tensor imaging

DTI has become the preferred modality for detecting and characterizing WM injury in mTBI. The WM consists of axonal bundles and water preferentially diffuses along the length of axons because of the barriers such as myelin, in other words, diffusion is anisotropic. DTI exploits the directional dependence of water diffusion in tissue to probe tissue microstructure. In contrast, water diffusion in GM is nearly isotropic. Anisotropic diffusion is described by a 3 × 3 symmetric diffusion matrix. All six independent elements of the diffusion matrix need to be determined for complete characterization of diffusion anisotropy. The diffusion matrix elements are determined by acquiring diffusion-weighted images (DWIs) in the presence magnetic field gradients. At least six DWIs are required, with noncollinear gradient directions. In practice more gradient directions are needed for robust determination of the diffusion matrix. In addition, MRI data have to be acquired without the application a diffusion gradient (commonly referred to as b0 images). Because DWI is sensitive to motion, it is important to ensure that DWIs are not corrupted by other motions, such as respiration. Therefore, DWIs are acquired using rapid sequences such as echo planar imaging or spiral sequences. Three principal diffusivities, λ_1_, λ_2_ and λ_3_ (also known as eigenvalues) and three eigenvectors are estimated from the DWIs. These are needed for generating the fiber tracts. By convention, λ_1_ has the largest value and is referred to as the longitudinal or axial diffusivity (AD). The average of λ_2_ and λ_3_ is referred to as the radial or transverse diffusivity (RD). The average of the three eigenvalues is referred to as the mean diffusivity (MD). The fractional anisotropy (FA), a dimensionless quantity, is the most commonly used measure of diffusion anisotropy and is derived from the three eigenvalues. FA varies from 0 to 1, with 1 representing perfect anisotropy and 0 representing complete isotropy. Depending on the WM tract, the value of FA is typically between 0.4 and 0.8 with the FA value of cerebrospinal fluid (CSF) close to 0.0. The GM which is mainly made of cell bodies has an FA value of <0.2. A lower FA value in response to injury is thought to represent compromised WM. However, this is not always true in the presence of crossing fibers within the imaging voxel. For example, if one of the crossing fibers is compromised while others are intact, an increased FA is expected [101]. Thus caution should be exercised in interpreting altered FA values. FA can be affected both by the axonal and myelin state and is therefore less specific about the pathologic underpinnings of the injury. It is generally thought that AD and RD reflect axonal and myelin integrity, respectively, and therefore may have better pathologic specificity than FA. However, recent DTI–histology correlative studies do not completely support this contention [102].

Another measure that can be derived from the DWIs is Shannon entropy (SE). SE is based on the information theory and is a measure of diffusion anisotropy. Unlike DTI, SE is derived from DWIs without assuming any diffusion model. SE is high for anisotropic structures such as WM and low for isotropic structures such as CSF. SE can reveal tissue anisotropy that may not be apparent on FA. SE appears to be more sensitive to axonal density and less affected by fiber orientation than FA. Based on correlation with histology, SE appears to detect axonal remodeling that occurs in response to neurological injury [103].

#### DTI acquisition & analysis

The acquisition parameters used for DTI can affect the most commonly used scalar variables, FA and MD [104–108]. The acquisition parameters include the number of gradient directions and the gradient scheme, the diffusion sensitization (commonly denoted by b), signal-to-noise-ratio (SNR), spatial resolution, field strength and even the scanner hardware and software. The number of gradient directions and spatial resolution affect FA, while diffusion sensitization (b) affects MD [108]. This dependence on the acquisition parameters makes it difficult to compare the diffusion results across different studies. Based on published studies, the reproducibility and repeatability of FA and MD improve with the number of gradient directions [106]. There is a general consensus that 30 gradient directions with a b value of approximately 1000 s mm^-2^ provides robust results.

The commonly used DTI analysis techniques can be classified into: whole brain histogram, region-of-interest (ROI), voxel-based and tractography. Each one of these methods has its own advantages and disadvantages. The whole brain histogram analysis is quite robust and simple. However, it does not provide any spatial information. The ROI analysis allows testing hypothesis that is specific to tract and is also useful for following individual subjects longitudinally. However, it requires *a priori* knowledge about region that needs to be examined and may introduce bias. Voxel-based analysis does not require *a priori* knowledge and is independent of any hypothesis. It provides information over the whole brain while retaining the spatial information. However, it could introduce errors due to nonlinear image registration and is also prone to local noise in the images. This method also requires image smoothing to reduce image misalignment errors from image registration. It is not easy to objectively determine the extent of required smoothing. TBSS (tract-based spatial statistics) is a popular technique that is representative of voxel-based analysis. It is most suitable for group analysis. In order to minimize registration errors, the fiber tracts are skeletonized. TBSS includes only major WM tracts and the information about the tract edges is lost. However, TBSS analysis includes stricter threshold to account for multiple comparisons and may have less statistical power than the ROI analysis. Tractography based analysis has become popular with the availability of multiple software packages. The diffusion measures can be determined for the whole tract or regionally along the length of the tract. The results of this analysis are dependent on the quality of tractography.

The SNR, voxel resolution and the complexity of the fiber arrangements and the tract curvature can significantly affect the quality of tractography [109]. Tractography results appear to be affected both by the algorithms and the software packages used. For example, in one study it was shown that both deterministic and probabilistic tractography algorithms (the most widely used algorithm) result in ‘systematically unreliable and clinically misleading information’ [110]. However, both algorithms work fairly well for the core of the tracts. A recent study compared qualitatively and quantitatively the results obtained with four different software packages: Brainance (Advantis Medical Imaging, Eindhoven, The Netherlands), Philips FiberTrak (Philips, Best, The Netherlands), DSI Studio [111], NordicICE (Nordic NeuroLab, Bergen, Norway) [112]. This study showed both qualitative and quantitative agreement for some tracts such as forceps major and forceps minor, but poor agreement for cingulum bundle, superior longitudinal fasciculus, inferior fronto-occipital fasciculus, corticospial tracts and superior longitudinal fasciculus. All these studies clearly point out the need for critical evaluation of the tractography based methods, especially at tract locations close to the cortex.

Finally the choice of the method for the analysis of the DTI should be chosen based on the purpose of the study.

#### Advanced diffusion imaging techniques

Majority of the published literature is based on the assumption that diffusion follows Gaussian distribution and that the DTI data are analyzed using a single tensor model. However, diffusion deviates from Gaussian behavior in brain which has a complex tract arrangement. Diffusion kurtosis imaging (DKI) [113] to some extent accounts for the non-Gaussian behavior of water diffusion. DKI also helps calculate axonal water fraction and assessment of axonal and/or myelin loss [114]. Conventional DTI is typically acquired at b = 0 and approximately 1000 s mm^-2^, with multiple gradient orientations. In contrast, DKI data are acquired at multiple b-values with multiple gradient orientations. To reduce the acquisition times, it is common to acquire DKI data with two nonzero b-values. Typically used b-values in DKI are approximtely 1000 cm^2^ s^-1^ and ∼2000 cm^2^ s^-1^. The commonly derived DKI measures are mean kurtosis and axial and radial kurtosis. The DKI and DTI measures are mathematically related [115].

Using conventional DTI, it is difficult to resolve crossing and kissing fibers. The inability to resolve crossing fibers makes the interpretation of diffusion measurements difficult. These problems can be overcome to some extent using advanced diffusion imaging. High angular resolution diffusion imaging (HARDI) is used to resolve crossing fibers (see, e.g., [116]). HARDI data are acquired using a larger number of gradient directions and orientations. HARDI is particularly useful for visualizing complex tract arrangement in brain. HARDI is typically acquired at multiple b values and large number of gradient directions. The orientation direction function is determined from the HARDI data for determining the tract direction. The anisotropy is generally expressed as diffusion anisotropy and generalized FA [116].

It is worth pointing that from the acquisition point of view DTI is a subset of DKI and HARDI. The DTI measures can be derived both from the DKI and HARDI data. The longer scan times are limitations of HARDI and DKI.

### Susceptibility-weighted imaging

Magnetic susceptibility, χ, is a basic material property that is a measure of the ability of an applied magnetic field to magnetize the material. Mathematically it can be represented by the equation M = χB, where M is the magnetization induced by the magnetic field B. Susceptibility is positive for paramagnetic and ferromagnetic materials and negative for diamagnetic substances, such as water. Susceptibility is a dimensionless quantity and different substances have different values. For example, the χ value of pure water is -9.05 × 10^-6^. In contrast, the χ values of deoxygenated and oxygenated red blood cells are -6.52 × 10^-6^ and -9.19 × 10^-6^, respectively. In comparison, ferritin loaded fully with 4500 ferric ions has a positive χ of 520 × 10^-6^. Tissues and biological substances have different susceptibilities. SWI exploits the differences in the susceptibility values between different tissues. SWI is most commonly used for visualizing veins and hemorrhage in the tissue. The principles and applications of SWI are recently reviewed [117]. It is one of the most sensitive techniques for visualizing microhemorrhage, thought to be an indicator of DAI. The sensitivity of SWI increases with the magnetic field strength.

### Magnetization transfer imaging

Tissue contains at least two types of water pools: mobile and tightly bound to macromolecules such as myelin. The MR signal mainly arises from the mobile water protons. Because of the short T2 relaxation time, the tightly bound water proton signal is very broad and cannot be directly detected on MRI. However, the presence of tightly bound water protons can be detected indirectly by transferring magnetization from the bound water protons to the mobile water protons using an off-resonance radio frequency (RF) pulse whose frequency is slightly different (∼ few kHz) from that of the mobile water proton frequency. Because of the large line width of the magnetic resonance signal from the bound water protons, this off-resonance pulse can excite the bound water protons without directly affecting the mobile water proton signal. This transfer of magnetization from one pool to another is referred to as magnetization transfer imaging (MTI). In a typical MTI experiment the MR signal is acquired with (M_on_) and without (M_off_) the off-resonance pulse. The MTR is calculated on a pixel-by-pixel basis as MTR = (M_off_ - M_on_)/M_off_. The MTR signal depends on the concentration of the bound water molecules, among others. Since myelin is the major macromolecular entity in brain, MTR is an indirect measure of myelin concentration. The MTR signal also depends on the amplitude and frequency of the off-resonance pulse. MTR is a semiquantitative technique that is affected by the experimental conditions. This limitation can be overcome by rapid and quantitative MTR techniques such as macromolecular proton mapping [118].

### Myelin water imaging

As indicated above, protons associated with myelin are much less mobile and have short T2 short relaxation time that varies from 10 μs to 1 ms and are difficult to detect with conventional MRI. However, water protons trapped between the myelin layers have a T2 relaxation value around 30 ms and can be detected using multi-echo sequences [119]. The myelin water fraction is defined as the ratio of water trapped between the myelin layers to the total tissue water and is a measure of myelin concentration. It is generally thought that this is an indirect measure of myelin.

### Ultrashort TE technique

As indicated above myelin has a very short relaxation time that is difficult to detect using conventional MRI. However, ultrashort echo time (UTE) techniques allow the detection of tissues with very short T2 relaxation times. The UTE techniques have been recently reviewed [120].

### Anatomical MRI

Hyperintense lesions in mTBI are sometimes observed on the T2-weighted FLAIR (fluid attenuation by inversion recovery) images. FLAIR sequence is used to suppress species with long T1 relaxation times, such as CSF in the brain. This is an excellent sequence for visualizing WM lesions, particularly in the periventricular lesions, in a number of neurological diseases such as multiple sclerosis [121].

Atrophy is generally thought to represent either loss of myelin and/or axons. MRI is an excellent modality for estimating regional, tissue-specific and whole brain atrophy. Atrophy is generally measured on high-resolution 3D T1-weighted images which show excellent GM–WM contrast. Cerebral atrophy is useful for following temporal changes in disease progression and response to treatment in neurological disorders such as multiple sclerosis [121] and Alzheimer's disease [122,123].

### Magnetic resonance spectroscopy

Magnetic resonance spectroscopy (MRS) exploits small differences in the nuclear resonance frequency as a result of the local chemical environment. These small frequency differences allow the detection and quantification of various molecules. Since biochemical changes precede anatomical changes, MRS has the potential to detect tissue pathology prior to visualizing on anatomical imaging. The four biologically most important nuclei for MRS are ^1^H (proton), ^13^C, ^23^Na and ^31^P. Of these nuclei, ^1^H has the largest abundance in tissue and highest MR sensitivity. In addition, ^1^H MRS (PMRS or simply, MRS) does not require additional hardware. Therefore, majority of the published studies focused on PMRS.

The major neurochemicals that can be detected with MRS are: n-acetylaspartate, (NAA) + N-acetylaspartylglutamate (NAAG), total creatine (Cr; creatine + phosphocreatine), total choline (Cho; has contributions from multiple molecules that include phosphorylcholine, glycerophosphorylcholine and choline plasmalogen, and a minor contribution from acetylcholine and choline), myoinositol (mI) and lipids. The resonant frequencies of NAA and NAAG are very close and cannot be easily separated. And also the concentration of NAAG is much lower than NAA. Therefore, in the literature, the combined peak is generally referred to as NAA. In addition to the above molecules, one can also detect lactate when it is present. Glutamate and glutamine overlap and difficult to resolve clearly at clinically used magnetic field strengths and the combined peak is referred to as Glx. Note that Glx may also have contributions from gamma-aminobutyric acid (GABA). Detection and quantification of molecules such as GAB require some type of editing. The MRS peaks are identified by their spectral location which is most commonly expressed as parts per million (ppm) and is independent of the magnetic field strength. For example, the resonance frequencies of NAA, Cr and Cho are 2.02, 3.0 and 3.2 ppm, respectively. Of all these neurochemicals, NAA perhaps attracted the greatest attention because it is considered to be a neuronal (cell bodies, axons and dendrites) marker in adult brain. Creatine is an indicator of total energy and intracellular metabolism. It is generally thought that Cr is unaffected by tissue pathology and is often used as an internal reference. However, a number of studies have shown that the level of Cr is affected by pathology. Choline reflects cell membrane metabolism. Elevated choline is a marker of cellular turnover and cellular proliferation. Myoinositol is considered to be a glial marker and precursor of phospholipid membrane constituents and its concentration is affected by the formation and breakdown of myelin. The concentration of lactate is fairly low in normal brain (CSF has a higher concentration of lactate than the brain parenchyma) and elevated lactate is a marker of anaerobic glycolysis.

MRS can be acquired either from one ROI at a time (referred to as single voxel MRS or SVS) or simultaneously from multiple voxels, referred to as MRS imaging (MRSI) or chemical shift imaging (CSI). SVS is simple to acquire and generally has superior MRS quality. MRSI allows visualization of spatial distribution within a large region and is technically a little more demanding than SVS and requires longer acquisition times. On modern MRI scanners both SVS and MRSI can be acquired routinely. For technical reasons, most frequently MRSI is acquired from a limited region in the brain. This requires spatial localization and the two most common sequences for spatial localization are point-resolved spectroscopy (PRESS) and stimulated echo acquisition mode (STEAM). The SNR with PRESS is twice that of STEAM. But STEAM can be acquired at shorter echo times than PRESS, thus allowing recovery of some of the lost SNR. It is also possible to acquire MRS from the whole brain without any localization, but is technically challenging. Since the neurochemical concentrations are several orders smaller than tissue water, techniques for water peak suppression are most commonly employed in MRS. However, more recently proton MRS techniques without water suppression are introduced [124].

MRS can be acquired either at short echo times (TE; ∼30 ms or less) and long echo times (∼140 or ∼270 ms). The advantage of short echo MRS is that neuochemicals with short T2 relaxation times such as mI and Glx can be visualized. However, not all the spectral peaks are resolved at short echo times, making it difficult to quantify. The disadvantage of long TE MRS is the difficulty in visualizing neurochemicals with short T2 relaxation times. However, in long TE MRS, the peaks show reduced overlap and the baseline is cleaner for more robust quantification. Long TE MRS is most commonly used to reduce the contamination from lipids (which have a shorter T2 relaxation times) and visualize lactate which overlaps with lipid peaks.

The two major issues with MRS are the relatively poor spatial resolution and long acquisition times. This is one reason why MRS has not gained popularity in routine clinical studies even though it predates MRI.

MRS is inherently a quantitative technique. The simplest way to quantify neurochemical concentrations is to express them as ratios relative to Cr. This assumes that Cr is unaffected by pathology which is not always true. There are number of software packages, both commercial and free for spectral quantification and determination of both absolute concentrations and as ratios. More details can be found in two recent reviews [125,126].

## Application of MRI to WM injury in mTBI

The application of the above-described MRI techniques to investigate WM changes in mTBI is reviewed below.

### DTI

The changes in WM on DTI are mainly assessed based on the altered FA and MD values relative to some control group or baseline values. Table 3 summarizes the published literature on the number of affected tracts in mTBI. This table is compiled based on 37 publications after 2011. As can be seen from this table, there is a lack of complete agreement on the location and number of affected WM tracts across different studies. For example, 24 out of 37 publications reported CC to be affected. This is followed by the IC; 10/37, superior longitudinal fasciculus (SLF; 10/37), CR (8/37) and anterior thalamic radiations (ATR; 7/37).

**Table 3.** **Frequency of white matter tracts affected in mild traumatic brain injury.**

**WM tract**	**Number of publications**
Corpus callosum (splenium 8; genu: 8; body: 2; regions not specified: 6)	24

Internal capsule	10

Superior longitudinal fasciculus	10

Corona radiata	8

Anterior thalamic radiations	7

Inferior longitudinal fasciculus	5

Frontal WM	4

Fornix	3

Cingulum	3

Centrum semiovale	2

Corticospinal tracts	2

These data are based on 37 publications since 2011.

WM: White matter.

A meta-analysis of the data based on 13 DTI studies (1980 through April 2012) based on the ROI analysis indicated that CC, particularly the splenium (SCC), was the most commonly affected WM structure in mTBI [77]. Another review of the published studies on sports-related concussion through February 2012 identified CC, IC, SLF as the structures most affected in mTBI [78]. It should be noted that the latter analysis is based on only eight studies. Another more recent meta-analysis of the voxel-based data generated in 17 studies (covering until 10 August 2015) determined that the SCC, ATR, left forceps minor and right SLF were the WM structures that were most commonly affected [91].

Some of the reasons for this discordance across different studies could be due to differences in the type of injury, DTI acquisition and analysis methods, post injury scan time, gender, sample sizes, use of improper comparison group and possible hemispheric asymmetry [30,60,63,107,127–129]. In addition, not all studies examined all of the WM structures. For example studies using ROI approach may have examined only a few selected tracts. As indicated earlier, the DTI results strongly depend on the post injury acquisition time [50]. For example, increased RD and reduced FA were observed within 72-h post injury that recovered after 2 weeks [59]. Similarly, changes in some DTI measures were observed around 24 h post injury time that were normalized by 3 months [30,61].

There is also disagreement on the direction of changes (increase or decrease) in the DTI measures in mTBI relative to baseline or the control group. Majority of the studies on mTBI reported lower FA and higher MD in WM [29]. Other studies reported decreased FA and increased MD at later postinjury time points. In contrast, other studies found increased FA even at later postinjury time points [7]. A meta-analysis revealed higher FA and lower MD in the acute phase, but opposite trend in the chronic phase [80]. In contrast, as reviewed elsewhere, some studies did not show differences in the DTI parameters at any postinjury time point [26]. Another meta-analysis of DTI data in the semi-acute phase of injury revealed that approximately equal number of studies reported elevated and reduced FA values in mTBI [107]. While increased FA and reduced MD are thought to represent cytotoxic edema, this interpretation was challenged [7]. As can be seen from Table 1, there is a large spread in the post injury times across different published studies. The effect of these various factors on the reported results makes an objective comparison of DTI results across different studies difficult. Despite the limited agreement across different studies, there is a general consensus that DTI is the most sensitive neuroimaging modality for detecting differences between mTBI and controls at a group level.

### Is DTI ready for managing individual patients?

Published studies clearly demonstrate considerable intersubject variations in FA that might reflect differences in anatomy, vulnerability to injury and injury mechanisms, among others [7,29,46,54,55,75]. Group analyses tend to mask differences among individual subjects. Detecting group differences in DTI parameters, while important, may be of little use for managing individual patients [26]. The TRACK-TBI study also pointed out the importance of considering individual subjects for predicting outcome in mTBI [42]. We are aware of only three publications that analyzed the data at an individual patient level [7,52,54]. For DTI to play an important role in patient management, it is critical to demonstrate the sensitivity of DTI in detecting changes in individual subjects. Using EZ-MAP approach that is shown to be robust for analyzing individual subject's data [54], Lipton *et al*. [7] reported ‘unique spatial patterns’ that are ‘attributable to interindividual differences in anatomy, vulnerability to injury and mechanism of injury’. Boiux *et al*. [52] reported that in patients with persistent post concussive symptoms, the individualized analysis has the potential for improving clinical management of these patients.

### Advanced diffusion imaging

The application of DKI to mTBI was recently reviewed [92]. So far only a limited number of studies used DKI for investigating WM changes in mTBI. For example using DKI, an association between mean kurtosis in various WM structures and attention, concentration, memory, learning and information processing was reported [130]. DKI was also reported be sensitive for following pathophysiological changes in the anterior and posterior capsule areas [131]. These authors also computed the DTI measures from the same data by dropping the terms containing higher order b-values. Their studies suggest that RD, AD, MD and FA may underestimate the extent of injury. However incorporating both DTI and DKI measures may improve assessment of tissue microstructure.

As indicated earlier, a single tensor model, by ignoring crossing and kissing fibers, may lead to misinterpretation of the results, but also may underestimate the extent of WM injury. Indeed a study using HARDI with 55 diffusion-gradient directions found WM damage to be more widely distributed than previously reported with DTI in Iraq and Afghanistan veterans with previous history of mTBI [132].

Injury to WM along the inter- and/or intrahemispheric pathways can disrupt brain connectivity [133]. Therefore, WM injury in mTBI was also investigated by probing the connectivity between different brain regions. The connectivity can be probed by combining DTI and resting state functional MRI (rfMRI; see [88] for a review). For example, functional connectivity between the two hemispheres in asymptotic mTBI subjects was reported to be reduced that may affect the structural integrity of the CC [134]. Altered structural and functional connectivity within the emotional and perception networks in the acute stage of mTBI was also observed [66]. Using machine learning, altered connectivity within inter- and intrahemispheric WM pathways that may be associated with diffuse axonal injury was demonstrated [135,136]. It is clear from this brief description that connectivity studies have the potential to yield a wealth of information on subtle WM injury that may not otherwise be apparent.

### Susceptibility weighted imaging

Microhemorrhages in WM may indicate DAI [8]. Microhemorrhagic lesions in the SCC and number of GM structures were observed at 6 months post injury on SWI [137]. In contrast, a prospective pilot study at 3T on college hockey players failed to detect microhemorrhages at 72 h, 2 weeks and 2 months after concussion [73]. In another study only about 9.8% amateur boxers showed more hemorrhagic lesions than controls on SWI [138]. However, this study was performed at 1.5T and it is possible that more hemorrhagic lesions could have been detected at higher field strengths. Overall, there is no complete agreement on the presence of microhemorrhagic lesions in mTBI.

The relationship between microhemorrhagic lesions and behavioral and cognitive deficits was investigated only in a few studies. A negative correlation between the microhemorrhagic lesions on SWI and cognitive impairment was reported [137]. However, it is not clear if this correlation is the result of hemorrhagic lesions in the SCC or GM. The multicenter TRACK-TBI study on 135 mTBI subjects showed that four hemorrhagic lesions early on after injury was predictive of outcome at 3 months [139]. However, these authors did not explicitly state if these are WM or GM lesions or a combination of both.

The total SWI lesion volume is suggested to be an indicator of injury severity [140]. While the SWI lesion volume could reflect injury severity, care should be exercised in relating the number of hemorrhagic lesions to the injury severity since the microbleeds could expand with time and become confluent that results in reduced number of bleeds [141]. Overall there is dearth of literature on WM SWI lesions in mTBI. Thus it is difficult to draw robust conclusions about the role of SWI lesions in WM injury.

### Magnetization transfer imaging

MTR is thought to be an excellent modality for probing myelin integrity. Based on both human and animal studies, myelin appears to be compromised in the initial phase of injury followed by recovery [97,142–144]. Demyelination compromises trophic support to axons and also releases neurite outgrowth inhibitor, Nogo, which has a negative effect on axonal growth and plasticity [97]. Despite its potentially important role, application of MTR to mTBI is limited. Earlier studies reported MTR changes in mTBI [38]. A recent prospective longitudinal study did not show MTR differences between mTBI and orthopedic controls either at approximately 24 h or approximately 3 months post injury time [30]. It is possible that 24 h is too early to observe changes in myelin and complete remyelination may have occurred by 3 months, explaining these negative results. Indeed a prospective study using myelin-water imaging of university hockey players reported decreased myelin water fraction at 2 weeks post injury that returned to the preseason level by 2 months after injury [67].

As indicated earlier, macromolecular proton fraction (MPF), a technique based on MTR, was used to investigate veterans who experienced blast mTBI [55]. This study indicated reduced MPF in CC, IC, SLF, superior parietal lobule and right precuneus compared with nonblast veterans. In addition, reduced MPF was also observed at the GM–WM junction that is consistent with DAI. It is however, unclear if reduced MPF completely reflects compromised myelin.

### Myelin water imaging

In spite of the ability of myelin water imaging to directly map the myelin water fraction, to the best of our knowledge, there is only a single study that applied this technique to concussion [67]. In this prospective and longitudinal study on two college hockey teams, reduced myelin water fraction was observed in multiple WM regions that included SCC, CR, right posterior thalamic radiation, left superior CR, left SLFC and left posterior limb of the IC at 2 weeks post injury followed by recovery by 2 months, suggesting transient myelin disruption. This study did not evaluate the behavioral changes in these players.

### Ultrashort TE imaging

UTE is capable of detecting macromolecules such as myelin with short T2 relaxation times. However, to the best of our knowledge, this technique so far has not been applied to mTBI.

### Atrophy

The number of published studies on WM atrophy is relatively small. Majority of the studies focused on whole brain atrophy and GM structures (see recent review [38]). WM atrophy was reported in the anterior cingulate WM and left cingulate gyrus isthmus WM [145]. In this study 28 mTBI patients and 22 controls were followed up to 1 year. These authors reported a correlation between atrophy of the left and right rostral anterior cingulum with changes in memory. They also reported a correlation between the left cingulate gyrus isthmus WM volume with PCS. WM atrophy in mTBI was also reported by others [146,147]. However, a longitudinal prospective study, using sophisticated tensor based morphometry, failed to observe atrophy in mTBI either at 24 h or 3 months relative to orthopedic injured controls [30]. It is possible that 3 months is too a short time to observe regional or global atrophy. Clearly longitudinal studies over a longer period of time are needed to assess the role of WM atrophy in mTBI.

### FLAIR

WM hyperintense lesions on FLAIR sequence were observed in mTBI [32]. In veterans with mTBI, it was shown that these lesions have an effect on verbal memory, independent of the presence of PTSD [148]. Unlike moderate and severe TBI, FLAIR hyperintense lesions are not that common in mTBI. When present, they are very nonspecific, and their significance is not known (perhaps they represent small vessel disease or residual shear-strain injury to WM). Further, it is not clear that these lesions are the result of mTBI or represent pre-existing condition.

### Magnetic resonance spectroscopy

MRS is second only to DTI as the most commonly used MRI-based technique in mTBI. Published MRS studies since 2011 are summarized in Table 4. This table does not include MRS articles that addressed only GM. Nor does this table include any review articles. The application of MRS to mTBI was reviewed elsewhere [31,38,81,149].

**Table 4.** **Summary of magnetic resonance spectroscopy results published on human mild traumatic brain injury since 2011.**

**Number of mTBI subjects/age in years and gender (F/M)**	**Number controls/age (F/M)**	**Field strength (T)**	**Postinjury time**	**Cross-sectional (X) or longitudinal (L)**	**MRS acquisition and analysis**	**TE (ms)**	**WM structure Absolute concentrations (AC) or ratios (R)**	**Main results**	**Ref.**
30/27.30 ± 9.52 (17/13)	30/29.41 ± 10.89 (15/15)	3	13.13 ± 5.90 days and 120.47 ± 1330 days	L (scanned at 2 time points)	2D CSI LC model	40	NI AC referenced to water	Concentrations of Cr and combined glutamate-glutamine signal were elevated in the mTBI group; partial normalization of these neurochemicals and NAA occurred in early days post injury and recovered during the semi-acute period	[34]

15/20.6 ± 1.2 (9/6)	15/20.4 ± 0.8 (8/7)	3	Mean 10.8 days	X	3D CSI Siemens software	135	SCC and GCC R	In genu both NAA/Cho and NAA/Cr ratios were significantly lower in mTBI group compared with controls; in GCC both NAA/Cho and NAA/Cr were significantly lower in mTBI compared with controls	[150]

28/20.3 ± 1.53 (15/13)	20/20.2 ± 0.83 (10/10)	3	11.4 ± 6.1 days	L	3DCSI jMRIU	135	SCC and GCC R	Reduced NAA/Cho and NAA/Cr in GCC and GCC regardless of number of mTBIs symptom resolution	[35]

With PCS (PCS+): 15/36 ± 1 (4/11) Without PCS (PCS-): 11/30 ± 11 (1/10)	12/(NI)	3	20 ± 9 days	X	3D CSI In-house software	NI	Global WM AC	PCS+ and PCS- did not differ in metabolite levels; lower WM NAA compared with healthy controls; MRS levels did not correlate with postinjury time	[9]

26/33 ± 11 (5/21)	13/33 ± 12 (5/8)	3	21 ± 14 days	X	2D CSI (3 interleaved slices) in-house software	35	Global WM AC (relative to phantom containing neurochemicals)	Mean Cr, Cho and mI levels in WM in mTBI were not different from controls; NAA levels in WM were significantly lower in patients than in controls; findings support hypothesis that, similar to more severe head trauma, mTBI also results in diffuse axonal injury, but that dysfunction rather than cell death dominates shortly after injury	[10]

11/24.6 ± 6.4 (3/8)	11/25.9 ± 5.7 (3/8)	3	3, 15, 30 and 45 days	L	Single voxel Philips provided software	144	WM at GM–WM junction R	Compared with controls, athletes showed increased NAA/Cr at 3 days post concussion, followed by decrease and subsequent normalization at 30 and 45 days post concussion; NAA/Cho decreased at 3, 15 and 30 days post concussion and normalized by 45 days; compared with controls, significant increase in Cho/Cr after 3 and 15 days and no differences were seen at 30 and 45 days	[36]

21/33.2 ± 12.4 (2/19)	22/31.5 ± 11.7 (2/29)	1.5	48.9 ± 16.0 h	X	Single voxel LC model	135	3 ROIs; bilateral frontal lobe in dorsolateral prefrontal area and upper brainstem (not pure WM) AC (arbitrary units referenced to water); ratios were used for statistical comparison	Reduced NAA/Cr in right and in NAA in left frontal lobe compared with controls; neurochemical changes correlated with cognitive decline and presence or absence of loss of consciousness in the acute phase of injury	[57]

ESA: 43/40.6 ± 17.3 (10/33) LSA: 33/37.6 ± 16.6 (10/23) Chronic: 27/40.1 ± 17.3 (9/18)	21/39.8 ± 18.0 (8/13)	3	5.44 ± 3.15 days, 37.0 ± 12.3 days, 195.3 ± 19.6 days	L	3D CSI LC model	135	CSV R; expressed relative to absolute Cr concentration	No statistically significant changes in NAA/Cr at any stage of mTBI compared with healthy controls; reduction in Cho/Cr at the LSA stage; no change in Cr at any stage of mTBI	[58]

62/30.4 ± 8.8 (19/43)	59/29.2 ± 9.0 (14/45)		25.5 ± 12.3 h and 97.9 ± 17.6 days	L	2 D CSI LC model	53	WM including CC R	No significant differences were found in any neurochemicals between mTBI and OI cohorts either at initial or follow-up scans	[30]

25/33.6 ± 11.2 (5/20)	NI	3	21.2 ± 14.3	X	2D CSI (3 interleaved slices) in-house software(?)	35	Global WM, GCC and SCC AC	Main purpose is to compare DTI, DKI with NAA; NAA may reflect TBI processes related to intra-axonal injury, as well as axonal degeneration in mTBI	[151]

This table only includes magnetic resonance spectroscopy studies on white matter.

LC model is a commercially available software package and jMRUI is a freely available software package for analyzing MRS data.

2D: Two-dimensional; 3D: Three-dimensional; AC: Absolute concentration; CC: Corpus callosum; Cho: Choline; Cr: Creatine; CSI: Chemical shift imaging (also referred to MRS imaging); CSV: Centrum semiovale; DKI: Diffusion kurtosis imaging; DTI: Diffusion tensor imaging; ESA: Early subacute; F: Female; GCC: Genu of corpus callosum; Glx: Glutamine + glutamate; L: Longitudinal; LSA: Late subacute; M: Male; mI: Myoinositol; NAA: N-acetyl aspartate; NI: Not indicated; OI: Orthopedic injured; PCS: Postconcussion symptoms; R: Reurochemical ratio; ROI: Region-of-interest; SCC: Splenium of corpus callosum; T: Tesla (magnetic field strength); TE: Echo time; X: Cross sectional.

Majority of the MRS studies report reduced NAA in the acute phase of injury [12,33,36,57]. A reduction in NAA/Cr and NAA/Cho in the subacute phase of injury in athletes with multiple concussions was also reported [35]. In a longitudinal study reduced NAA and elevated Cr and Glx in WM around day 13 post injury in the mTBI group was observed that was followed by partial recovery during the semi-acute period [34]. This study also noted that premorbid intelligence predicts the degree of recovery of the neurochemicals indicating that the degree of recovery depends on the biological factors underlying intelligence. In a cross-sectional study on concussive injured athletes continued neurochemical alterations were observed even when the clinical symptoms have resolved [152]. This is consistent with other reports using advanced neuroimaging techniques [33,88,153,154]. In another study in acute phase neurochemical changes were observed only in subjects with LOC [57]. However, it appears that the ROI's in this study represent a mixture of both GM and WM and therefore the reported association may not be attributed completely to WM injury. In an interesting study on a small number of subjects, the WM levels of NAA, Cr, Cho and mI were compared in mTBI subjects with and without PCS [9]. These authors reported changes in these neurochemicals levels in PCS positive, but not in PCS negative subjects. This suggests that MRS has the potential to differentiate between PCS positive and PCS negative subjects. In another study, the regional metabolic levels were correlated with neuropsychological data [58]. These authors also investigated if neurochemical changes observed in the early subacute phase (within 10 days post injury time as defined by these authors) were predictive of neurocognitive outcome at 6 months post injury. This study demonstrated an association between Cr levels in the centrum semiovale in the early subacute phase and the automated neuropsychological assessment metrics scores in the chronic phase. In addition, these authors also reported decreased Cho/Cr in the late subacute phase. These results suggest that MRS measures have the potential to serve as diagnostic and prognostic markers in mTBI. Consistent with other studies, the neurochemical levels returned to the normative values in the later stage of injury.

There is no complete agreement about the changes in the neurochemical levels in mTBI. For example a few studies failed to observe any changes in NAA levels [30,34,152]. A prospective study on an mTBI cohort failed to observe changes in NAA/Cr, Cho/Cr and mI/Cr either, at approximately 24 h or approximately 3 months post injury [30]. Based on the published literature, it is likely that 24 h post injury is too early for metabolic changes to occur and any possible neurochemical changes may have disappeared by 3 months [36].

A review of Table 4 explains why it is very difficult to generalize the conclusions reported across different MRS studies. Many studies did not specify the WM regions from which the spectra were acquired even though it is known that metabolite levels depend on the brain area (see e.g. [45,81]. The type of MRS acquisition varied across different studies. Some studies employed single voxel technique while others used 2D and 3D MRSI using different echo times. A few studies expressed the neurochemicals as ratios while others expressed as absolute concentrations. Ratios would not allow assessment of whether the altered numerator or denominator is responsible for the observed neurochemical changes. For example, many of the conclusions about NAA reduction are based on measured NAA/Cr ratios. This assumes that the Cr levels are not affected by mTBI, which is a questionable assumption. A few studies that reported absolute concentrations used a variety of methods to quantify neurochemicals. The MRS data were acquired at different postinjury times even though the neurochemical levels were shown to change with postinjury time [10,58]. Another problem is the placement of the ROIs. Because of the relatively poor spatial resolution, many of the ROIs included part of GM that makes it difficult to attribute the observed changes only to WM pathology. Finally majority of the studies were based on a relatively small sample size.

## MRI & pathology correlation

Histology is the gold standard for evaluating mTBI-induced pathology. However, histologic evaluation is not ethically justified in human mTBI. This is where noninvasive neuroimaging such as MRI could play a central role. While MRI is an excellent radiologic modality for visualizing and following pathology, its pathologic specificity is somewhat limited. In mTBI the commonly observed MRI abnormalities in WM are: altered diffusion measures that include FA, RD, AD, MD on DTI; hypointense lesions on SWI; atrophy (seen on anatomical MRI); reduced MTR on MTI; changes in the myelin water fraction on myelin water imaging and macromolecular fraction imaging; and altered neurochemical concentrations (NAA, Cr, Cho and mI) on MRS. In the absence of histologic confirmation, our interpretation of the MRI changes is mainly based on preclinical models of mTBI. Since DTI is the most commonly used imaging modality in mTBI, majority of the imaging–pathology correlation studies focused on DTI.

There is significant literature on the MRI–pathology correlation in different diseases. However this correlation may depend on the disease type. There are only a handful of studies that investigated the MRI–histology correlations in preclinical models of mTBI. A summary of the published MRI–histology correlation studies is provided in Table 5. Almost all the published studies agree that various pathological substrates contribute to the observed changes in the DTI parameters and establishing one-to-correspondence between the DTI measures and histology is not straight forward. Majority of the studies suggest that axonal injury (including demyelination) mainly contributes to the observed alterations in the DTI measures [11,137,141,155–159]. However, some authors argue that inflammation also contributes to the altered DTI measures [160,161]. There is some agreement that RD is a measure of myelin integrity [142,157,159]. AD is thought to represent axonal damage [155]. However, there is some disagreement about this interpretation [142,160,161]. In summary, based on the published studies it is not straightforward to relate changes in DTI measures to pathology since they are affected by coherence of the fiber tracts, inflammation, gliosis, demyelination and axonal compromise [145].

**Table 5.** **Preclinical studies since 2011 that include immunohistochemistry and neuroimaging.**

**Animal species and sample size (n)**	**Injury/surgical procedure and description**	**Postinjury time**	**Brief summary of methods and affected WM structures**	**Behavioral tests and results**	**Histology**	**Ref.**
Female Wistar rats 10 (TBI: 5; sham: 5)	Controlled cortical impact/craniotomy	2 months	Both *in vivo* and *ex vivo* DTI (15 and 30 gradient directions for *in vivo* and *ex vivo*, respectively) at 7T ROI analysis CC	–	GFAP, MBP, MAP2, SMI31, SMI32 stainings Decreased MBP and GFAP; SMI 31 and SMI 32 unaltered	[160]

Male Wistar rats 10 (TBI: 5; Sham: 5)	Impact–acceleration model (Marmarou)/midline scalp incision Severity not mentioned	30 min, 1, 2, 4 and 5 h	*In vivo* DTI (6 and 21 gradient directions) at 7T; ROI analysis CC	–	Hoechst and MBP stainings: No evidence of fiber organization Silver staining: Fiber disorganization in traumatized CC	[155]

Sprague–Dawley albino rats 16 (4 per group at 4 time points)	mTBI Impact–acceleration (Marmarou) model/not indicated	4 and 24 h, 3 days and 7 days	*In vivo* and *ex vivo* DTI: TBSS analysis (field strength and number of gradients not indicated) CC SWI voxel-wise analysis left CC	–	APP staining: Corresponding to the SWI and DTI showing axonal injury in CC	[140]

Male C57Bl/6 mice 18 (rcTBI at 3 different time points: 12; sham: 6)	Mild repetitive closed-skull TBI (rcTBI) using electromagnetic stereotaxic impact Device/midline incision Two concussive impacts within a 24-h period to exposed skull		*In vivo* DTI (6 gradient directions) at 4.7 T; ROI analysis CC and EC	–	APP staining Only occasional APP-positive swellings were detectable in injured mice Iba-1 and silver staining Positive at 7 days post injury (microglial activation and axonal injury)	[156]

Male Sprague–Dawley rats 48 (1 atm: 20; 1.5 atm: 11; sham: 17)	mTBI; lateral fluid percussion injury at pressure of 1.0 or 1.5 atm/craniotomy	Day 15 (?)	*Ex vivo* DTI (42 gradient directions) at 7T ROI analysis CC, IC, EC and Ci	1.0 atm FPI: no vestibulomotor, motor, or spatial learning and memory deficits 1.5 atm FPI: transient motor dysfunction and transient deficit in motor coordination seen Spatial learning and short-term memory dysfunction	Silver staining Increased silver impregnation observed in the CC. Ci, IC and EC at 1.5 atm FPI APP staining Decreased myelination in CC ipsilaterally at 1.5 atm FPI Iba-1 and GFAP increased inflammation in CC at both 1.0 and 1.5 atm FPI even at 2 weeks injury	[11]

Male Sprague–Dawley rats 21 (single mTBI: 7; rmTBI: 7; sham: 7)	mTBI; CCI/craniotomy single mTBI to right cortex rmTBI (bilateral) Initial injury to right cortex followed by a second injury to the left cortex after 7 days	60 days post injury	*Ex vivo* DTI (6 gradient directions) at 11.7T ROI analysis CC and Ci	–	Luxol Fast Blue staining Increases in right and left CC area and midline CC width in rmTBI group Electron microscopy of CC: ↑ g-ratio (↑ axon caliber & ↓ myelin thickness in rmTBI) Indicating WM ultrastructure compromise	[157]

Male Sprague–Dawley rats 28 (7 groups: 4/group; group1: control; groups 2, 3 and 4: blunt trauma; groups 5, 6 and 7: blast trauma)	Mild blunt and blast trauma with a custom-built device/none	1, 4 and 7 days	*In vivo* DTI (21 gradient directions) at 3 T Three-dimensional fiber tracking CC	–	H&E staining Increased voids in the extracellular matrix following blunt and blast compared with controls Both injuries reduced nearest neighboring distance of fibers at day 1 and 7	[162]

Male Sprague–Dawley rats 11 (CCI: 8; sham: 3)	CCI over left primary forelimb SIFL/craniotomy	1 and 3 h and 2, 7 and 14 days	*In vivo* DTI (30 gradient directions) at 7T ROI analysis CC	Sensorimotor function Foot fall scores in affected limb on day 1; improved on days 2 and 7; returned to normal on day 14 Forelimb asymmetry test Present on days 1 and 2	Nissl stain Some tissue cavitation and loss at impact region and surface laterally on ipsilateral side Fluro-Jade staining Number of degenerative neurons increased ipsilaterally	[163]

Male Ferrets 11	Mild CCI injury to left hemisphere somatosensory cortex/craniotomy (?)	24–28 h	*In vivo* and *ex vivo* DTI (30 gradient directions) at 7T Voxel-wise analysis followed by ROI placement that included GM and WM near the injury site Evidence of WM abnormalities	–	Tuj1 and MAP2 stainings: Reduced neuronal elements in the cortex ipsilateral to the lesion Iba-1 staining: Enlarged microglia with extensive processes on ipsilateral side compared with contralateral hemisphere MOG Decreased myelinated fibers in upper layers of neocortex	[164]

Male Sprague–Dawley rats 45 (MRI: 10; other analysis: 4 h: 5; day 1: 5; day 3: 5; day 5: 5)	mTBI closed head injury; modified Marmarou's/?	4 h and 1, 3 and 5 days	*In vivo* DTI (46 gradient directions at 7T ROI analysis: CC	–	GFAP staining No change in CC	[165]

Female Wistar rats 30 (mTBI + MVM: 15; controls: 15)	mTBI closed head injury (modified Marmarou weight drop)/scalp shaved	1 and 8 days	*In vivo* DTI (15 gradient directions) and MTR at 7 T ROI for DTI and voxel-wise for MTR CC	–	SMI3I staining Increased axonal injury on days 1 and 8 in CC MBP staining Patchy demyelination in CC Iba-1 Increased microgliosis in CC	[166]

Female Wistar rats n = 45 (injured: 40; uninjured controls: 5)	mTBI Modified Marmarou weight drop closed head injury/scalp shaved	1, 10, 20 and 30 days	*In vivo* DTI (15 gradient directions) and MTR at 7T DTI based on ROI and MTR based on voxel-wise analysis CC, AC, OT, EC, ST and CP	–	SMI31 staining Significant neurofilament loss on days 1 and 10 and returned to baseline level on day 30 in CC MBP staining: insignificant loss of myelin signal on days 1, 10 and 30 in CC Iba1 staining Increased on day 1 and reached maximum on days 10 to 20 in CC GFAP Increased over 30-day time course in CC TUNEL staining Positive and reached highest value maximum on day 20 in CC	[159]

Male Sprague–Dawley rats 30 (MRI paired with histology: 12; cytokine analysis: 12 (6 injured, 6 uninjured; controls for histology: 6)	mTBI closed head Maryland model/malar processes exposed via infraorbital incisions	72 h	*In vivo* DTI (42 gradient directions) at 7T Voxel-wise followed by ROI GCC, SCC, Fi and IC	Exploratory behavior Injured animals explored less	MBP expression Significantly decreased in IC, SCC and Fi Iba1 staining Increasing trend in microglial response to injury	[142]

Male wild-type C57BL/6 10 (DTI: n = 10 Seriology/PCr: 5–8)	Repetitive mild closed head injury (rmCHI)/none (?) 7 consecutive injuries over 9 days	1 week after last hit	*Ex vivo* DTI (30 gradient directions) and SWI at 4.7T CC, Fimbria and capsular WM	–	Iba-1 and CD68 staining Increased gliosis at 24 h and 1 week in WM	[161]

Male Sprague–Dawley rats 18 (Group 1: 6 with widespread increases in RD in CC: Group 2: 12 with normal RD)	mTBI: CCI/craniotomy	1 and 3 h and 1, 2, 7 and 14 days	*In vivo* DTI (30 gradient directions) and QSM at 7 T ROI analysis CC	Forelimb placement (cylinder) and foot fault tests Increased front limb asymmetry and number of forelimb foot faults with recovery on day 14 in injured rats more in group 1	Black gold staining Myelin loss (white matter damage) in CC (impacted hemisphere)	[167]

Studies on only WM are included in this table. Since in animals classification of TBI as mTBI is somewhat ambiguous, some important studies are included even when the publications do not explicitly classify the injury as mTBI.

?: Indicates that explicit details are not provided in the publication; AC: Anterior commissure; AD: Axial diffusivity; atm: Atmosphere; AO: Apical oblique; APP: Amyloid precursor protein; BS: Basal shift; CC: Corpus callosum; CCI: Controlled cortical impact; Ci: Cingulum; CP: Cerebral peduncle; DTI: Diffusion tensor imaging; EC: External capsule; EPM: Elevated plus maze; FA: Fractional anisotropy; Fi: Fimbria; J: Joule (unit of energy); LISW: Laser-induced shock wave; MCW: Medical College of Wisconsin; MD: Mean diffusivity; mFPI: Mild fluid percussion injury; MRS: Magnetic resonance spectroscopy; MTR: Magnetization transfer ratio; MVM: Mild ventriculomegaly; MWM: Morris water maze; OT: Optic tract; PLP: Proteolipid protein; PSI: Pounds per square inch; QSM: Quantitative susceptibility mapping; rcTBI: Repetitive closed skull TBI; RD: Radial diffusivity; rmCHI: Repetitive mild closed; rmTBI: Repeated mTBI; TAI: Traumatic axonal injury; WDI: Weight drop injury.

The lesions seen on SWI are shown to correlate with hemorrhage on histology [140,161]. However, care must be exercised in interpreting the SWI lesions since it is sometimes difficult to directly compare the Prussian reaction, used for identifying hemorrhage, with heme degradation [141,161].

MTR is generally thought to reflect the state of myelin. Based on a recent study, MTR appears to be more affected by astrogliosis and microgliosis than demyelination [159]. MTR changes also depend on the offset frequency of the off-resonance pulse [159]. For example, the MTR with off-resonance frequency of 3.5 ppm reflects both axonal and myelin integrity while MTR with offset frequency of 20 ppm mainly reflects astrogliosis. Thus it appears difficult to attribute MTR changes to a single pathologic component.

To the best of our knowledge there are no studies that reported correlation between MRS, MWF with pathology in mTBI.

As can be seen from this brief summary of the MRI–pathology correlative studies, MRI measures reflect different pathologic components, depending on the time and type of injury, experimental conditions. Robust correlation, between MRI findings and neuropathology requires the use of appropriate animal models of mTBI. However, there is a lack of consensus on what represents mTBI in animal models [168–171]. The commonly used neurological severity score that is a composite of behavioral and motor functions and righting reflexes for grading severity of TBI [168,172] is of limited use in in experimental models of mTBI. Even with all these limitations, DTI, MRS, MTR and SWI measures are very sensitive imaging modalities for detecting mTBI-induced pathology.

## Conclusion & future perspective

Based on the above review it is clear that the multimodal neuroimaging has diagnostic and prognostic values in detecting and evaluating WM changes in mTBI. These MRI-based modalities are also able to detect ongoing pathology even when the subjects are asymptomatic. However, MRI measures also show wide variation across different studies. In order to properly compare results from different studies to develop a coherent picture about the MRI-detected pathology and its correlation with cognitive and behavioral deficits, it is essential to standardize the acquisition and analysis methods. Similarly there is a need to standardize the behavioral and cognitive measures. Majority of the publications are based on cross-sectional studies with relatively small sample size. Cross-sectional studies have lower statistical power relative to longitudinal studies. Longitudinal studies also provide important information about the evolution of pathology. For example we know relatively little about the long-term consequences of mTBI. Given the heterogeneity of the injury, age, gender and other variables, studies based on a small cohort may not allow for robust conclusions. This requires studies on large and well-characterized cohorts. However, it may not be easy to recruit adequate number of subjects needed for statistically robust results by a single center. Recruiting large cohorts requires multicenter design with well-standardized protocol and rigorous quality control in place. It is also important to include both genders since some of the published studies show gender effect on pathology and behavioral deficits. It is equally important to consider age as a covariate in the analysis of the results since many of the MRI measures are age dependent. There are a few studies that demonstrate hemispheric asymmetry in mTBI. Future studies should explicitly state the side of the hemisphere in their results. A major problem in the field of mTBI is the lack of appropriate diagnostic criteria and the absence of a biomarker. As pointed out by Manley and Maas [1], there is a critical need to incorporate imaging information for diagnosing mTBI. Finally there is a need to develop preclinical mTBI models, particularly using large animals, to interpret the observed changes in MRI measures on a rational basis.

## Limitations of this review

This review is based on publications after 2011 and includes only references published after 2011. Thus original references are not cited. However, references to recent reviews are provided, whenever possible, so that the readers can have information about the original work. While there are multiple neuroimaging modalities other than MRI, for this review which focuses on only WM injury, other imaging modalities such as CT, PET, single-photon emission computed tomography, near-infrared are of limited use. Finally, this study did not try to separate blast from mechanical mTBI. This study also did not separate single and repetitive mTBIs.

**Executive summary**

- Noninvasive neuroimaging techniques, particularly MRI-based diffusion tensor imaging and magnetic resonance spectroscopy, have considerably improved our understanding about WM injury in mild traumatic brain injury (mTBI). They also enhanced the diagnosis and prognosis and help follow pathophysiological changes in mTBI.
- Many MRI-derived white matter injury measures correlated with cognitive and neurobehavioral deficits, which suggests a predictive role for this neuroimaging technology.
- There is no complete agreement on neuroimaging results across different studies. A problem with most of the published MRI studies in mTBI are the variability in injury, postinjury time at which studies are performed and not accounting for the site of impact.
- Most of the published studies are either cross-sectional or are based on a small sample size. There is need for controlled studies on large cohorts.
- Majority of the published studies are based on single center that makes it difficult to validate results across different centers.
- Classification of mTBI is based on Glasgow Coma Score whose sensitivity is relatively poor in diagnosing mTBI. There is a critical need to incorporate imaging information for diagnosing mTBI.
- Lack of standardization in the acquisition and analysis techniques across centers prevents an objective comparison across different studies.
- While group analysis is important, there is a need to investigate the role of imaging in managing individual patients.
- Imaging–pathology correlation is not always consistent. There is some urgency in performing controlled preclinical studies for a rational interpretation of the MRI results.
- Despite these limitations neuroimaging is indispensable for the management of mTBI.
